# Microbiological quality of beef, mutton, and water from different abattoirs in the Eastern Cape Province, South Africa

**DOI:** 10.14202/vetworld.2020.1363-1371

**Published:** 2020-07-18

**Authors:** Philisani Ncoko, Ishmael Festus Jaja, James Wabwire Oguttu

**Affiliations:** 1 Department of Livestock and Pasture, University of Fort Hare, Private Bag X1314, Alice, 5700, Eastern Cape Province, South Africa; 2 Department of Agriculture and Animal Health, University of South Africa, Florida Campus, Johannesburg, 1709, South Africa

**Keywords:** contamination, foodborne pathogens, hygiene, meat spoilage, water quality

## Abstract

**Background and Aim:**

Abattoir processes from skinning, evisceration, to chilling usually lead to meat contamination by foodborne pathogens. Hence, continual microbial surveillance of slaughter carcasses by veterinary public health officials is key to preventing contamination and outbreak of meat-related foodborne diseases. This study was conducted to determine the *Enterobacteriaceae* count and aerobic plate count (APC) and to detect *Escherichia coli* and *Salmonella* spp. in meat and water from selected slaughter facilities.

**Materials and Methods:**

Retrospective data (n=100) collected in 2017 by the Provincial Veterinary Department of the Eastern Cape Province from abattoirs and prospective survey data of meat (n=50) collected in 2018 from abattoirs in the Eastern Cape Province were utilized in this study. APC and *Enterobacteriaceae* were enumerated from the samples. In addition, *Salmonella* and *E. coli* were isolated from samples using selective media.

**Results:**

The APC in both retrospective and prospective studies for all samples ranged between 2 and 4.50 log CFU/cm^2^; similar counts of 2-4.00 log CFU/cm^2^ were recorded for *Enterobacteriaceae*. No significant difference (p>0.05) for APC and *Enterobacteriaceae* count across all meat types was noted. *Salmonella* and *E. coli* were detected in 50% of beef. *E. coli* was not detected from mutton, but *Salmonella* was found in 66.7%. Moreover, 91.7% of the water samples had *E. coli*, but none had *Salmonella*.

**Conclusion:**

The levels of *Enterobacteriaceae* and APC observed in meat satisfy regulatory conditions outlined by the Department of Agriculture, Forestry and Fisheries, South Africa and show that meat produced from these abattoirs is of acceptable microbial quality. However, the quality of water used in the abattoirs does not meet the requirements set by the government, and contributes to contamination of meat produced in the abattoirs under study. Therefore, we recommend that sources of water be continuously investigated to eliminate or reduce the risk of contamination of meat processed in the abattoirs.

## Introduction

Meat is an outstanding source of protein in human diets, but because of its biochemical composition, it is highly susceptible to microbial contamination. The contamination of meat usually leads to severe spoilage and foodborne infections [1]. Meat-borne pathogens are easily transferred to meat from the animal gastrointestinal tract, the environment, and the meat handler’s hands, especially in poor sanitary conditions. Recent studies have indicated that consumers are now searching for healthier and nutritious meat [2].

A large proportion of the South African population is reliant on mutton, chicken, beef, and pork as their source of protein, predisposing them to infection if contaminated [3,4]. Consuming contaminated meat is the principal transmission route for foodborne disease. Pathogens, including *Campylobacter*, *Staphylococcus*, *Escherichia coli*, *Salmonella*, and *Enterococci*, are listed as the top five food pathogens worldwide [5]. These pathogens have been identified as the causative agents of millions of infection and mortality globally [6]. In developing countries, around 33% of the population are affected by foodborne illnesses yearly [7]. Moreover, an estimated 230,000 or 40% of the infections due to non-typhoidal *Salmonella enterica* occur in children, resulting in mortality [8]. In the WHO subregion, AFR D and E circa 2012, around 10,200 cases of Shiga toxin-producing *E. coli* food-related illnesses were reported [9].

Hundreds of livestock for both domestic and international meat markets are processed in abattoirs in South Africa. Process stages, including stunning, skinning, scalding, evisceration, and chilling are sensitive critical control points (CCP) for microbial contamination prevention.

Microbial testing of meat after slaughter ensures that hygiene breaches are corrected in record time. However, this is seldom done in some provincial abattoir, necessitating the need for this study [10].

Even though a few studies have reported the health risks associated with consuming meat and meat products in some provinces in South Africa, there are few studies on the microbiological quality of meat and water in abattoirs in the ECP. Hence, the objective of the current study was to evaluate the microbiological quality of meat and water in different abattoirs in the province.

## Materials and Methods

### Ethical approval

The University of Fort Hare Ethics Committee (MUC551SNCO01) approved all the protocols that were used in the experiments carried out in this study.

### Description of the study site

The Eastern Cape Province is the second-largest province in South Africa (SA), with an estimated population of 6,522,700 based on the mid-year population estimates of 2018 [11]. The ECP is one of the provinces with a high number of livestock. The number of cattle, sheep, and goats in the province is estimated to be 3.139 million, 6.615 million, and 2.085 million, respectively [12]. The province’s vast landmass caters for various farming systems ranging from communal farming to commercial farming. These farming systems practice different production systems, including extensive, semi-intensive, and intensive production systems. The ECP has more than 70 abattoirs that cater for both red meat and poultry slaughter. These abattoirs are distributed across six district municipalities of the Eastern Cape, and include: Hani, O.R Tambo, Joe Gqabi, Alfred Nzo, Amathole, and Sarah Baartman district municipalities.

#### Collection of retrospective data (RD) and prospective survey (PS)

A retrospective data (RD) of the microbial survey of meat from March 2017 to December 2017 collected by the Provincial Veterinary Department, Veterinary Public Health Unit, were included in this study. The RD consisted of a microbial count for both beef and mutton. A prospective survey (PS) was also carried out in different abattoirs from the six districts such as Alfred Nzo, Amathole, Chris Hani, Joe Gqabi, Sarah Baartman, and O.R Tambo District Municipalities (Table-1). In the PS, samples of beef, mutton, and water were collected from January 2018 to November 2018.

**Table-1:** Abattoir location, classification of facility and species slaughtered at facility.

Number	Abattoir Location	Coordinates	Classification of facility	Species slaughtered
1	Stutterheim	32.5885° S, 27.4321° E	High throughput	Poultry
2	East London	33.0292° S, 27.8546° E	Rural throughput	Poultry
3	Stutterheim	32.5885° S, 27.4321° E	Rural Throughput	Poultry
4	Indwe	31.4803° S, 27.3440° E	Low Throughput	Cattle and Sheep
5	East London	33.0292° S, 27.8546° E	High Throughput	Sheep, Cattle and Pigs
6	Maclear	31.0638° S, 28.3345° E	Rural Throughput	Sheep, Cattle and Pigs
7	Tsolo	31° 19’ 0” S, 28° 45’ 0” E	Rural Throughput	Cattle and Sheep
8	East London	33.0292° S, 27.8546° E	High Throughput	Cattle, Sheep, Game and Pigs
9	Elliot	31.3130° S, 27.8370° E	High throughput	Cattle and Sheep
10	Matatiele	30.3621° S, 28.8014° E	Low Throughput	Cattle, Sheep and Pigs
11	Queenstown	31.9127° S, 26.9597° E	High Throughput	Cattle and Sheep
12	Adelaide	34.9285° S, 138.6007° E	High Throughput	Game
13	Komga	32.5906° S, 27.8839° E	Low Throughput	Sheep and Cattle
14	Barkly East	30.9691° S, 27.5907° E	Rural Throughput	Cattle, Sheep and Pigs
15	Komga	32.5906° S, 27.8839° E	Low throughput	Cattle, Sheep and Pigs
16	Molteno	31° 24’ 0” S, 26° 33’ 0” E	Low Throughput	Sheep and Pigs
17	Adelaide	34.9285° S, 138.6007° E	Low Throughput	Cattle, Sheep and Pigs
18	Aliwal North	30° 42’ 0” S, 26° 42’ 0” E	Low Throughput	Cattle, Sheep and Pigs

E- East, S- South, High throughput (<50 units to >100 units), Low throughput (Cattle 20 units, sheep and goats 40 units and Pigs 30 units), Rural throughput (Maximum of two units/ per day) 1 unit = 1 cattle or 6 sheep or 5 pigs or 4 ostriches

### Sampling of carcasses and collection of samples

The sampling was performed following the completion of carcass dressing before the commencement of chilling. Carcasses were sampled once a month. The samples were taken halfway through the slaughter day and on the sampling day to acquire samples that are representative of the factory’s daily throughput. The swabs from each carcass were sufficiently labeled and placed in a sterile container containing 100 mL of sterile diluent and transported to the laboratory at 4°C between 8 and 24 h.

Swabs were moistened before the collection of samples with the use of sterile maximum recovery diluent 0.1% peptone and 0.85% NaCl for a minimum of 5 s. The swabs were taken from the carcass by swabbing diagonally, horizontally, and vertically for not <20 s with the use of a sterile 100 cm^2^ template and as much pressure as possible. The surface area for swabbing was approximately 100 cm^2^. Swabbing of different carcasses was done in the following manner: Bovine (neck, brisket, flank, and rump) first and then ovine (flank, lateral thorax, brisket, and breast).

### Microbial count

#### Aerobic plate count (APC) and Enterobacteriaceae

According to the International Organization for Standardization recommendation (ISO 21528-2, 2009), 25 g of each sample was utilized for culturing. The sample was poured in a stomacher bag. The weighed sample was then added to 9 mL of buffer peptone water (Lasec, South Africa), giving a 1:10 dilution. The samples in the buffer peptone water were poured into a stomacher bag (Bag mixer^®^DOA 20550). The stomacher bag with the sample was placed in a bag mixer machine and mashed for 3 min. Afterward, 0.1 mL of the test sample was transferred into two Petri dishes with the use of a sterile pipette.

APC was acquired by including 0.1 mL of the suspension and a series of ten-fold dilutions (10^−1^, 10^−2^, and 10^−3^) in duplicate onto plate count agar plates ([PCA] Lasec, South Africa). The prepared plates were inverted and then placed in an incubator at 30±1°C for 72 h±3 h. Following incubation, bacteria colonies on plates were counted with the use of a colony counter-digital machine (Lasec, South Africa). For *Enterobacteriaceae* enumeration, 1 ml of the sample was placed on the violet-red bile glucose agar, and a series of ten-fold dilutions (10^−1^, 10^−2^, and 10^−3^) was duplicated. The plates were incubated for 24 h at 37°C. Colony counting was performed, and colonies that appeared pink to red or purple were chosen and subjected to biochemical confirmation tests (oxidase test and glucose fermentation test) following international standards (ISO 21528-2, 2004).

#### E. coli

*E. coli* was identified in accordance with the International Organization for Standardization guidelines (ISO 16649-2, 2001). Twenty-five grams of the samples were emptied in a stomacher bag (Bag Mixer^®^DOA 20550) and added to 225 ml of peptone buffered water. The stomacher bag with the sample was then placed in a bag mixer machine and mashed for 3 min. Afterward, 0.1 ml of the test sample was transferred into the tubes with the use of a sterile pipette. Then, the mixture was incubated at 37°C for 24 h [13]. The identification of *E. coli* was performed in accordance with the International Organization for Standardization guidelines, with the use of the most probable number technique (ISO 16649-2 2003). The tubes exhibiting gas production were recorded as positive, and a loop-full from each positive gas tube was transferred to a separate tube with MacConkey Broth (Oxoid, UK). *E. coli* confirmation was achieved by observing the gas production and acidification during growth in MacConkey Broth (Oxoid, UK). The positive results were streaked onto tryptone bile glucuronic agar (TBX agar, Oxoid, UK), and the plates were incubated at 37°C for 24 h. The pink colonies were counted using a colony counter-digital machine (Lasec, South Africa) and further subjected to indole and catalase tests.

#### Salmonella spp

Twenty five grams (25 g) of each sample was weighed and emptied into a stomacher bag (Bag Mixer^®^DOA 20550), to which 225 ml of peptone buffered water was added. Each sample was processed in accordance with the International Organization for Standardization methods (ISO 6579, 2002). The stomacher bag was placed in a bag mixer machine to homogenize the sample. The contents of the stomacher bag were emptied into a 250 ml flat-bottom flask, which was already marked for identification. For the pre-enrichment stage, the flask was placed in an incubator (Labcon model: South Africa) at 37°C for 24 h. Following incubation, 0.1 ml of the pre-enriched broth was emptied onto Modified Semi-Solid Rappaport-Vassiliadis (MSRV) Agar (MSRV; Merck, Darmstadt, Germany) and incubated at 44°C for 24 h. After 24 h, a loopful was taken from MSRV and streaked onto Xylose Lysine Deoxycholate (XLD) Agar (XLD; Merck, Darmstadt, Germany) plates and inoculated at 37°C for 24 h. The colony count was performed following the completion of incubation in accordance to the ISO methods (ISO 6579, 2002).

#### Water analysis

The counts for APC and *E. coli* were carried out using the surface spread technique (on MacConkey Agar [Oxoid, Basingstoke, UK]). For water, APC and *E. coli* were obtained by pouring 100 ml of the sampled water onto a filter paper (pore size 0.44 μm) to trap as well as isolate bacteria. Following filtration, the filter paper was then placed in a Petri dish, holding the PCA (Oxoid Basingstoke, UK), cooled and incubated at 35°C for 48 h. Further isolation of *E. coli* was performed using eosin methylene blue (EMB) agar (Oxoid, Basingstoke, UK). The Petri dishes were incubated for 24 h at 35°C. Greenish metallic blue colonies in EMB agar were regarded as presumptive for *E. coli*. Biochemical tests, for example, indole and catalase tests, were utilized to confirm the *E. coli* isolates. Indole Kovac’s reagent was clear and light yellow in color (ISO 4883, 2014; ISO 21528-2, 2004).

### Statistical analysis

Data on microbiological count were first transformed to log (base 10) prior the analysis using Excel worksheet for easy comparison and were presented as means, standard deviation, and standard errors of the mean. The effects of month, meat type, and season on water microbial count were assessed with the use of the generalized linear model procedures of the statistical analysis system (SAS, 2009). Significant differences among group means were tested with the use of least significant differences, and the statistical significance level was set at p≤0.05. The results for the microbiological counts were also compared with the National Directorate Veterinary Quarantine and Public Health (VPN15 and 16) standards for meat and water [13,14].

## Results

### Microbial count in RD and PS data sets

The results from the RD indicate that the APC for beef ranged from 2.51 to 4.32 log CFU/cm^2^, and the *Enterobacteriaceae* count for beef was between 2.58 and 3.91 log CFU/cm^2^. The APC for mutton ranged from 2.48 to 4.38 log CFU/cm^2^, and the *Enterobacteriaceae* count for mutton was between 2.48 and 3.45 log CFU/cm^2^ (Tables-2 and 3). Water values for APC and *Enterobacteriaceae* were 1.71-1.91 and 1.58-1.80 CFU/ml, respectively (Table-4).

**Table-2:** Retrospective microbial count of beef from different abattoirs in the Eastern Cape Province.

Date	Location	Abattoir	Number of samples	Site swabbed	APC (log CFU/cm^2^)	*Enterobacteriaceae* (log CFU/cm^2^)
24 May 2017	Aliwal North	K	2	NA	4.00	ND
					4.28	ND
24 May 2017	Barkely East	L	2	NA	2.95	2.88
					ND	ND
12 June 2017	East London	H	4	Neck	ND	ND
				Rump	ND	ND
				Flank	ND	ND
				Brisket	4.01	ND
03 July 2017	Adelaide	J	4	Neck	ND	3.28
				Rump	ND	ND
				Flank	3.71	ND
				Brisket	3	2.90
06 August 2017	Stutterheim	M	4	Neck	ND	ND
				Brisket	3.52	3.18
				Flank	2.96	2.94
				Rump	ND	ND
26 September 2017	Matatiele	G	9	Carcass	2.59	2.61
				Carcass	2.88	2.84
				Carcass	3.06	2.83
				Carcass	ND	ND
				Carcass	ND	ND
				Carcass	3.00	2.95
				Carcass	ND	ND
				Carcass	2.69	2.58
				Carcass	ND	ND
26 September 2017	East London	H	3	Brisket	ND	ND
				Flank	2.84	2.74
				Neck	ND	3.00
10 October 2017	Elliot	F	2	Rump	2.58	2.69
				Neck	ND	2.99
18 October 2017	Indwe	D	3	Neck	2.65	2.95
				Flank	2.83	2.81
				Rump	ND	ND
24 October 2017	East London	E	3	Flank	ND	ND
				Rump	ND	ND
				Neck	ND	3.91
27 November 2017	Indwe	D	2	Brisket	4.32	ND
				Rump	4.01	ND
09 December 2017	Elliot	F	5	Rump	ND	ND
				Brisket	4.09	3.18
				Neck	2.70	ND
				Flank	2.51	ND

APC – Aerobic Plate Count. ND – Not Detected. VPN15 standards for meat, Aerobic plate count (3.5 log CFU/cm^2^- 5.0

log CFU/cm^2^), Enterobacteriaceae (1.5 log – 2.5 log CFU/cm^2^), *E. coli* (0 log – 1 log CFU/cm^2^), *Salmonella* Absent/25 g, NA=Information missing

**Table-3:** Retrospective microbial count of mutton and lamb from different abattoirs in the Eastern Cape Province.

Date	Location	Abattoir	Number of samples	Site swabbed	APC (log CFU/cm^2^)	*Enterobacteriaceae* (log CFU/cm^2^)
07 March 2017	King Williams Town	C	4	Flank	3.12	2.61
				Brisket	ND	ND
				Neck	2.51	ND
				Rump	ND	2.48
29 March 2017	King Williams Town	C	4	Neck	4.07	2.72
				Brisket	4.20	2.95
				Flank	4.38	ND
				Rump	3.40	3.17
29 March 2017	East London	H	4	Rump	3.50	2.52
				Neck	3.88	3.28
				Flank	4.15	3.45
				Brisket	2.77	3.06
13 June 2017	East London	E	4	Rump	2.64	ND
				Brisket	2.61	3.03
				Flank	2.61	2.61
				Neck	4.26	3.02
10 September 2017	Komga	O	4	Rump	ND	ND
				Neck	2.69	ND
				Brisket	3.67	3.29
				Flank	3.33	2.84
18 September 2017	Barckely East	L	2	Carcass	2.52	2.68
					ND	ND
17 October 2017	Komga	O	4	Neck	3.39	2.63
				Rump	ND	ND
				Flank	3.32	2.84
				Brisket	2.89	2.62
23 October 2017	Adelaide	J	6	Rump	ND	ND
				Brisket	3.30	3.18
				Neck	ND	ND
09 December 2017	Komga	O	4	Rump	2.48	ND
				Brisket	ND	ND
				Neck	2.64	2.49

APC – Aerobic Plate Count. ND – Not Detected. VPN15 standards for meat, Aerobic plate count (3.5 log CFU/cm^2^- 5.0 log CFU/cm^2^), Enterobacteriaceae (1.5 log – 2.5 log CFU/cm^2^), *E. coli* (0 log – 1 log CFU/cm^2^), *Salmonella* Absent/25 g

**Table-4:** Microbial count of tap water used by different abattoirs in the Eastern Cape Province.

Date	Location	Abattoir	No. of Samples	Sample type	APC (log CFU/ml)	*Enterobacteriaceae* (log CFU/ml)
29 July 2017	Enoch Sontonga	S	2	Tap water	1.76	1.59
				Tap water	1.71	ND
17 October 2017	Komga	O	3	Tap water	1.91	1.58
				Tap water	ND	ND
				Tap water	1.91	1.80
27 November 2017	Indwe	D	2	Tap water	ND	1.79
				Tap water	ND	ND

APC – Aerobic Plate Count, ND – Not Detected

In the PS, no significant differences in APC across all meat were noted. Equally, no significant difference (p>0.05) for *Enterobacteriaceae* for all meat types was noted. Specifically, the highest APCs for beef, mutton, and water were 3.54 log CFU/cm^2^, 4.14 log CFU/cm^2^, and 3.2 log CFU/cm^2^, respectively. The highest *Enterobacteriaceae* counts for beef, mutton, and water were 2.96 log CFU/cm^2^, 3.9 log CFU/cm^2^, and 3.2 log CFU/cm^2^, respectively (Table-5). Both *Salmonella* and *E. coli* were detected in 50% of beef. Even though there was no *E. coli* on mutton, *Salmonella* was detected in 66.7% of mutton samples. None of the water samples was positive for *Salmonella*, but 91.7% were positive for *E. coli* (Table-6). The mean APCs for beef, mutton, and water were 2.2 (SD: ±0.74), 3.0 (SD: ±0.49), and 1.9 SD: ±0.26), respectively (Table-7).

**Table-5:** Prospective microbial count Aerobic plate count and *Enterobacteriaceae* on beef, mutton and water.

Date	Meat	APC (log CFU/cm^2^)	*Enterobacteriaceae* (log CFU/cm^2^)
18 February 2018	Mutton	0	0
	Mutton	3.98	0
	Mutton	2.52	2.96
20 February 2018	Mutton	2.71	2.56
	Mutton	4.3	0
	Mutton	2.51	0
05 February 2018	Mutton	0	0
	Mutton	4.14	0
	Mutton	3.48	0
	Mutton	3.54	0
27 February 2018	Water	1.87	0
	Water	0	3.2
19 February 2018	Water	2.09	1.76
18 February 2018	Water	2.08	1.83
	Water	2.08	1.9
02 June 2018	Water	2.05	1.54
	Water	1.69	0
31 January 2018	Water	1.97	0
	Water	2.93	0
13 March 2018	Water	1.95	1.86
	Water	2	2
04 November 2018	Water	1.51	0
	Water	2.36	0
	Water	3.2	0
19 February 2018	Water	0	1.76
18 February 2018	Water	2.08	1.83
	Water	2.08	1.9
02 June 2018	Water	2.05	1.54
	Water	1.69	0
31 January 2018	Water	1.97	0
	Water	2.93	0
13 January 2018	Water	1.95	1.86
	Water	2	2
04 November 2018	Water	1.51	0
	Water	2.36	0
18 February 2018	Beef	3.54	2.5
	Beef	3.98	3.9
	Beef	2.52	2.96
	Beef	2.3	0

VPN15 standards for meat, Aerobic plate count (3.5 log CFU/cm^2^- 5.0 log CFU/cm^2^), *Enterobacteriaceae* (1.5 log – 2.5 log CFU/cm^2^), *E. coli* (0 log – 1 log CFU/cm^2^), *Salmonella* Absent/25 g

**Table-6:** *Salmonella* and *Escherichia coli* detection in beef, mutton and water.

Species	*Salmonella*			*Enterobacteriaceae*		
	
	+(%)	-(%)	±SD	+(%)	-(%)	±SD
Beef	50	50	0.58	50	50	0.58
Mutton	33.3	66.7	0.49	ND	100	0.00
Water	ND	100	0.00	8.3	91.7	0.28

+ Positive – Negative, SD-Standard deviation, ND-Not detected

**Table-7:** Aerobic plate count and *Enterobacteriaceae* count in beef mutton and water.

Species	APC		*Enterobacteriaceae*	
	
	μ±SE	#	μ±SE	#
Beef	2.2±0.74	Ns	1.0±0.74	Ns
Mutton	3.0±0.49	Ns	0.4±0.49	Ns
Water	1.9±0.26	Ns	1.0±0.26	Ns

μ-Mean, SE-Standard error, APC-Aerobic plate count.

# significance, Ns-Not significant, ** significant at P≤0.05

## Discussion

### APC and *Enterobacteriaceae*

Mishandling of meat has been identified as among the major public health issues. Sanitation and hygiene are essential factors that contribute to meat contamination at the abattoir. Studies have indicated a direct relationship between sanitary conditions at abattoirs and the level of APC and *Enterobacteriaceae* and *E. coli* counts of raw meat [15,16]. However, in the current study, the results for the retrospective and prospective survey demonstrate that all the count for APC and *Enterobacteriaceae* was within the acceptable limits as stipulated in the South African policy on the microbiological monitoring of meat, process hygiene, and cleaning [13]. The South African policy specifies the acceptable limits for APC as (i) acceptable (3.5 log), (ii) marginal (≤5.0 log), and (iii) unacceptable (>5.0 log) and *E. coli* as (i) acceptable (a) if counts are ≤1 CFU/cm^2^ (0 log); (ii) marginal (m) if counts are ≤10 CFU/cm^2^ (1 log); and (iii) unacceptable (u) if counts are >10 CFU/cm^2^] [13].

Similar results were reported in Spain, Switzerland, Korea, New Zealand, and Uganda, where APC and *E. coli* were reported to be between 2 and 4.5 log CFU/cm^2^ [1,17-19]. On the contrary, other studies conducted in Ghana and Egypt had higher APC, *E. coli*, and *Enterobacteriaceae* counts, ranging from 5.7 to 6 log CFU/cm^2^, respectively [20,21]. This result shows that sufficient hygiene measures were in place at the abattoirs involved in this study, which result in the low numbers of bacterial count in meat. Nonetheless, the occurrence of *E. coli* is of concern as some strains like *E. coli* O:157: H7 associated with the production of Shiga toxins have been reported to be the cause of foodborne illness in humans [22-24].

Other pathogroups, including enterotoxigenic *E. coli* and enteroaggregative *E. coli*, diffusely adherent *E. coli*, and enterohemorrhagic *E. coli*, are regularly transmitted to humans through the consumption of contaminated water and meat [25-27]. In the present study, no significant difference (p>0.05) was noted for *Enterobacteriaceae* in both beef and mutton. The result of APC is similar to that of *Enterobacteriaceae* for all types of meat. Hence, we suspect that the minimal contamination took place during animal slaughter. Studies conducted in the USA and Latvia also reported no significant difference (p>0.05) in *Enterobacteriaceae* and APC beef, minced meat, breaded pork, smoked meat products, chop, different types of sausages, aspic, and liver pate [28-30].

### Salmonella

This study found *Salmonella* in 50% of beef and mutton. In studies conducted in South Africa, Turkey, Denmark, and Egypt, the prevalence of *Salmonella* was found to be 3%, 5%, 10%, and 33%, respectively [31-33]. However, there was no *Salmonella* detected in another South African study [34]. *Salmonella* is still among the top five foodborne pathogenic bacteria causing remarkable health problems to consumers. In low- and middle-income countries, the lack of an epidemiological surveillance system makes it hard to assess the incidence of salmonellosis in both human and animals [33]. Hence, the recovery of *Salmonella* from meat is a public health hazard, with extreme consequences for children, older adults, people with HIV/AIDS, and pregnant people [35]. The rate of *Salmonella* found in this study proposes that meat acquired from the sampling area pose a public health hazard to consumers and hence compromises the quality of meat [13], thus highlighting the need for abattoirs to review their hygiene systems with the objective of identifying risk factors for *Salmonella* cross-contamination.

### Water

*E. coli* and other coliform bacteria are ideal indicators of water quality [36]. The South African government standards on water stipulate zero (0 log CFU/100 ml) for *E. coli* or coliforms and 100 CFU/ ml in water for total plate counts [14]. However, 91.7% of water samples in the current study tested positive for *E. coli*, *Enterobacteriaceae*, and APC ranged from 1.0 to 3.20 log CFU/ml. *Enterobacteriaceae* consists of a group of Gram-negative bacteria known to cause infections such as urinary tract infections, meningitis, cystitis, pneumonia, wound sepsis, and bacteremia [37]. Hence, the presence of *Enterobacteriaceae* poses remarkable public health risk if found in food and water. Such risk could further be worsened if the bacteria already have antimicrobial resistance, such as multidrug resistance. Water in prior studies had been linked to increase in bacterial count and could contribute to further spread of contamination of carcasses [10,38]. The result of bacterial counts in meat in the current study closely mirrors those of water; hence, we hypothesize that the water used for carcass washing could be responsible for carcass contamination.

## Conclusion

This study found that even though the beef and mutton from the abattoir were of good, acceptable microbial quality, the presence of *E. coli* in water compromised the quality of meat generated in such abattoirs. The observed levels of *E. coli* have the potential to predispose the meat to contamination with pathogenic *E. coli*. Because the biochemical composition of meat makes it ideal for the rapid proliferation of bacteria once contaminated, it is important that the sources of water used in abattoir be continuously investigated to eliminate or reduce the risk of contamination of meat processed in the abattoirs. Meat hygiene must also be maintained throughout the value chain for meat and meat products to protect the consumer’s health. Therefore, regular microbial testing during singeing, blasting and chilling of meat as part of monitoring the product while in production, in line with the principles of HACCP, should be implemented. Moreover, training of abattoir workers is needed to enhance hygienic skills as well as improve microbial meat quality. Remedial actions aimed at preventing the transmission of *Salmonella* either from the environment or through fecal contamination should be implemented.

## Authors’ Contributions

PN carried out the research and wrote the manuscript. IFJ designed the study, supervised the research, and edited the manuscript. JWO edited the manuscript and made a useful contribution to the study design. All authors read and approved the final manuscript.
